# Proteomics Analysis of Brain Tissue in a Rat Model of Ischemic Stroke in the Acute Phase

**DOI:** 10.3389/fnmol.2020.00027

**Published:** 2020-02-27

**Authors:** Fei Zheng, Yan-Tao Zhou, Yi-Fu Zeng, Tao Liu, Zhao-Yu Yang, Tao Tang, Jie-Kun Luo, Yang Wang

**Affiliations:** ^1^College of Electrical and Information Engineering, Hunan University, Changsha, China; ^2^Laboratory of Ethnopharmacology, Institute of Integrative Medicine, Xiangya Hospital, Central South University, Changsha, China

**Keywords:** proteomics, brain tissue, acute ischemic stroke, LC-MS/MS, omics

## Abstract

**Background**: Stroke is a leading health issue, with high morbidity and mortality rates worldwide. Of all strokes, approximately 80% of cases are ischemic stroke (IS). However, the underlying mechanisms of the occurrence of acute IS remain poorly understood because of heterogeneous and multiple factors. More potential biomarkers are urgently needed to reveal the deeper pathogenesis of IS.

**Methods**: We identified potential biomarkers in rat brain tissues of IS using an iTRAQ labeling approach coupled with LC-MS/MS. Furthermore, bioinformatrics analyses including GO, KEGG, DAVID, and Cytoscape were used to present proteomic profiles and to explore the disease mechanisms. Additionally, Western blotting for target proteins was conducted for further verification.

**Results**: We identified 4,578 proteins using the iTRAQ-based proteomics method. Of these proteins, 282 differentiated proteins, comprising 73 upregulated and 209 downregulated proteins, were observed. Further bioinformatics analysis suggested that the candidate proteins were mainly involved in energy liberation, intracellular protein transport, and synaptic plasticity regulation during the acute period. KEGG pathway enrichment analysis indicated a series of representative pathological pathways, including energy metabolite, long-term potentiation (LTP), and neurodegenerative disease-related pathways. Moreover, Western blotting confirmed the associated candidate proteins, which refer to oxidative responses and synaptic plasticity.

**Conclusions**: Our findings highlight the identification of candidate protein biomarkers and provide insight into the biological processes involved in acute IS.

## Introduction

Stroke is a leading health issue, with high morbidity and mortality rates worldwide (Raffeld et al., 2016). This neurological disease leads to permanent physical and neurological disability. Nearly 80% of all strokes are ischemic stroke (IS; Liu et al., 2010). IS incidence is induced by the sudden blockage of cerebral arteries, followed by a reduction in blood flow. After IS, the brain is unable to obtain enough oxygen and nutrition, resulting in damage to and death of neural cells (Adams et al., 1993). Despite progress in pathophysiology, the mechanisms underlying the occurrence of acute IS remain poorly understood because of heterogeneous and multiple factors. More potential biomarkers are urgently needed to reveal the deeper pathogenesis.

To date, several studies exploring potential biomarkers following IS have been published (Zhou et al., 2017; Makris et al., 2018). However, a lack of biomarkers with sufficient bioinformatics prediction hinders the clinical prognosis (Muñoz et al., 2018). Moreover, these studies merely focus on peripheral blood-based biomarkers, which provide little help for discovering novel therapeutic targets and elucidating sophisticated molecular mechanisms in brain (Rai et al., 2002; Laskowitz et al., 2009; Devaux, 2017; Misra et al., 2017). IS induces remarkable cellular and physiological changes within cerebral infarction and thus causes neurological disorders. These occurrences of pathophysiological information originate from brain tissue rather than blood. Additionally, due to the existence of the blood-brain barrier, the key proteins related to pathogenesis from peripheral blood may not entirely reflect the pathophysiological features in brain following IS. Therefore, more potent biomarkers from brain tissue with better predictive values are required for diagnosis and treatment.

Recently, a new “omics” approach has been developed that is promising for systemic analysis. The method can precisely target more candidate biomarkers and comprehensively discover biological processes. This technique may lead to improvements in diagnosis and treatment and hence better prognosis for IS (Cevik et al., 2016; Sun et al., 2019; Wesley et al., 2019; Zhu et al., 2019).

Among the omics analysis tools, proteomics enables us to investigate the overall protein status of various samples in the pathological disease in terms of quantitative state (Lindsey et al., 2015). To date, several proteomics profiles have suggested that brain ischemia impairs putative neuroprotective functions (Yang et al., 2014). For example, Sharma et al. (2015) provided a panel of protein candidates related to the pathophysiological features for diagnostic assays in serum after stroke. However, the absence of novel and sufficiently differential proteins impedes the full illumination of the underlying molecular mechanisms in the acute phase of IS (Catanese et al., 2017). Moreover, the discovery of ischemic biomarkers merely from plasma cannot deeply explain the proteomic characteristics (Whiteley et al., 2011; Chen et al., 2018). Consequently, investigation of the proteomics of acute IS is better performed using brain tissue samples.

**Figure 1:**
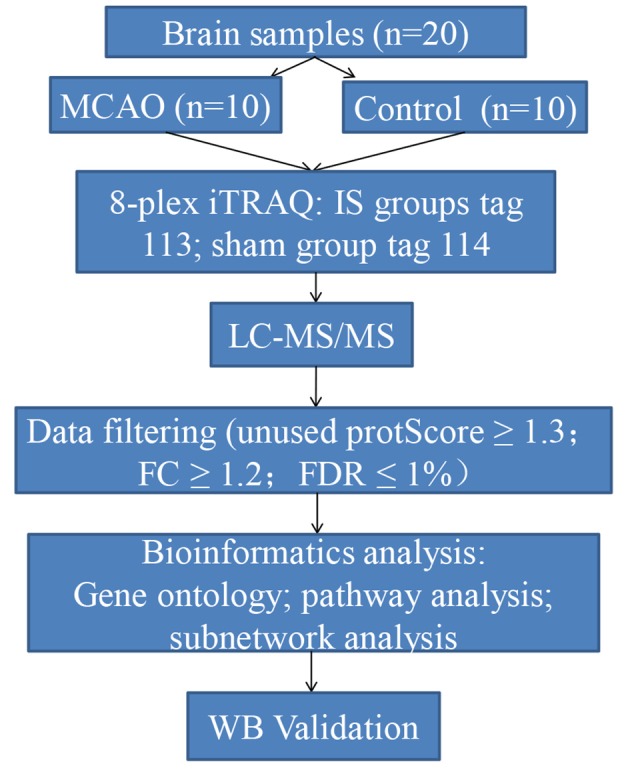
Flow chart of the proteomics analysis in this study.

In this study, we aimed to identify aggregated candidate biomarkers in the rat IS brain tissues using an iTRAQ labeling approach coupled with liquid chromatography-tandem mass spectrometry (LC-MS/MS). In addition, bioinformatrics analyses including GO, KEGG, DAVID, and Cytoscape were used to establish proteomic profiles and to explore the underlying mechanisms of acute IS. Furthermore, Western blotting was conducted for further verification. Our findings offer novel biological insights into the proteomic mechanisms of acute IS to guide its effective clinical diagnosis and treatment. A diagram illustrating the steps involved in this study is shown in Figure 1.

## Materials and Methods

### Ethics Statement

The Animal Research Committee of the Xiangya Hospital of Central South University approved all procedures and protocols in compliance with institutional guidelines and care guidelines (Animal Research Committee approval number: 201603112). All animal experimental procedures were conducted and verified by the Animal Ethics Committee of Central South University. At the endpoint of the experiment, all rats were humanely sacrificed with an intra-peritoneal euthanyl injection according to the Care guidelines.

### Experimental Animals and IS Mode Induction

The male Sprague–Dawley (SD) rats (acclimatized for at least 7 days, 220–240 g) were purchased from the Laboratory Animal Centre of Central South University. Rats were housed with ad libitum access to food and water. They were kept at 40–60% relative humidity, 21–25°C room temperature, and under a 12 h light/dark cycle. After adaptive feeding, the rats were randomly allocated into two groups as follows: sham group (*n* = 10) and model group (*n* = 10).

Rats from the model group were produced by transient intracranial middle cerebral artery occlusion (MCAO), as previously described (Wang et al., 2013). Briefly, the animals were placed in a supine position under anesthesia induced *via* injection of pentobarbital sodium (50 mg/kg). They were subjected to MCAO, with the right common carotid artery (CCA), the right external carotidartery (ECA), and the right internal carotid artery (ICA) exposed and isolated. The CCA was inserted into the origin of the middle cerebral artery (MCA) with a 1% poly-L-lysine coated 4–0 nylon monofilament suture (Beijing Cinontech Company Limited, Beijing, China). The Circle of Willis was reached with the suture. Afterward, reperfusion was accomplished by withdrawal of the MCA-suture after 2 h of occlusion.

Reperfusion was allowed after the suture had been withdrawn 24 h after MCAO. The sham group underwent all of the surgical procedures except MCAO occlusion. Physiological saline was given for both groups under the same conditions. All surgeries were performed in a sterile environment with body temperature maintained at 37 ± 0.5°C throughout the operation process. After surgery, the rats regained consciousness, showing no signs of discomfort, severe motor deficit, or seizures. According to our experiment, the successful rate of MCAO was >85%. The rats were then sacrificed. The brain tissues around infarction areas were removed rapidly and frozen at −80°C for further analysis.

### TTC Staining

To determine the brain infarct volume, 2,3,5-triphenyltetrazolium chloride (TTC) tests were performed. At 24 h following MCAO, brain tissues were removed and stored at −20°C for 20 min. Next, the brain tissues were sectioned into 2-mm thick coronal sections. They were incubated with 2% TTC at 37°C for 30 min. The volume of white area, which represents infarction, was measured by an image analysis system (Image-ProPlus 6.0, Media Cybernetics, MD, USA). The calculation formula is as follows: Infarct volume % = (white infarct area × thickness)/(whole slice area × thickness) × 100%.

### Proteomics Experiment and Proteomics Data Analysis

Proteins from samples were extracted by powering with liquid nitrogen and were further dissolved with lysis solution. The supernatant was collected after the cell lysate had clarified and been centrifuged at 12,000 rpm for 20 min at 4°C.

From each sample, 200 mg of protein was digested with alkylate and trypsin overnight at 37°C. Subsequently, labeling of peptides with their respective tags was performed according to the manufacturer’s instructions for the iTRAQ Reagents 8-plex kit (AB Sciex, Foster City, CA, USA), which were as follows: model group: tag 113; sham group: tag 114.

The iTRAQ-labeled peptides were subjected to strong cation exchange (SCX) chromatography by an HPLC system (Shimadzu LC-20AD HPLC pump system, Shimadzu Corp, Kyoto, Japan). The digested peptides were reconstituted with buffer A (20 mM HCOONH4, PH 10) and loaded onto a Gemini-NX C18 SCX column (3 μm, 2 × 150 mm, Phenomenex) with buffer B (20 mM HCOONH4 80% acetone PH 10). The flow rate was 200 μl/min. The elution absorbance (214 nm/280 nm) was monitored when the fractions were collected. Eluted fractions were pooled into 24 fractions and were concentrated by vacuum centrifugation. Each final fraction was reconstituted in 50% trifluoroacetic acid for LC-MS/MS.

LC-MS/MS analysis of the peptides was performed using a Q Exactive Mass Spectrometer (Thermo Fisher Scientific, Waltham, MA, USA) and identified with a Thermo Dionex Ultimate 3000 RSLC nano-system. The peptide mixture was separated from the PepMap C18 RP column (2 μm, 75 μm × 150 mm, 100 A) in buffer A (0.1% formic acid) and in buffer B (84% CAN in 0.1% formic acid) with a linear gradient of 4–90% at a constant flow rate of 300 nl/min over 65 min. Each peptide was dissolved into 0.1% formic acid and 2% acetonitrile (ACN) solution.

MS data were acquired by using a data-dependent top 20 mode and high-energy collisional dissociation (HCD) method with the scan range of the mass detector set to 350–1,800 m/z, which was chosen according to the most abundant precursor ions. The dynamic exclusion was set at 40 s, and the automatic gain control (AGC) was 3E6 to determine the target value. The under-fill ratio was defined as 0.1% to specify the minimum percentage of the target value.

Next, the raw data were obtained for protein identification and quantitation by ProteinPilot Software (AB Sciex, Foster, CA, USA; version 5.0.1) on the basis of the UniProt rat database. The search parameters were set as follows: iTRAQ-labeled 8-plex peptide for sample type, trypsin for digestion enzyme, and biological modifications for ID focus. The confidence threshold cut off (unused protScore) ≥1.3 or peptide confidence = 95%, as well as fold change (FC) ≥1.2 were considered to identify the differentially expressed proteins (DEPs) for further bioinformatics analysis. These data were filtered based on a false discovery rate (FDR) ≤1% for protein identification.

### Data Availability

All MS data were deposited in the online PRIDE Archive with the dataset identifer PXD006437 (http://www.ebi.ac.uk/pride/archive/login; Username: reviewer58335@ebi.ac.uk; Password: IXAJht8T).

### Bioinformatics Analysis

To annotate the potential functions of DEPs, Gene Ontology (GO) mapping and Kyoto Encyclopedia of Genes and Genomes (KEGG) pathways analysis was performed using DAVID Bioinformatics Resources v6.8^1^. Additionally, we used the STRING software (STRING v10.0) to predict protein-protein interaction (PPI) networks composed of key proteins and their interactions. The networks were constructed and analyzed by using Cytoscape 3.5.1. Pathways were considered statistically significant at a *p*-value ≤ 0.05.

### Western Blotting

The cerebral tissues from each group (*n* = 6/group) were collected on ice-cold to be lysed. The lysates were clarified by centrifuging at 12,000 *g* and 4°C for 10 min. The protein concentration was determined with a bicinchoninic acid (BCA) protein assay kit (Well Biotechnology Company, Changsha, China). The proteins from the supernatant of each sample were subjected to 10% sodium dodecyl sulfate polyacrylamide gel electrophoresis (SDS-PAGE) and then transferred onto polyvinylidene fluoride (PVDF) membranes. The membrane was blocked in a 5% milk-Tris-buffered saline and Tween 20 (TBST) solution for 1 h at room temperature. The membranes were incubated with primary antibododies, including rabbit polyclonal antibody against SOD1 (#10269-1-AP, 1:500, Proteintech) and SYN1 (#20258-1-AP, 1:2,000, Proteintech). The rat antibody against β-actin (#20536-1-AP, 1:5,000, Proteintech) was used as the internal reference. Subsequently, the membranes were incubated with an appropriate secondary antibody at room temperature. Proteins were visualized by using ECL Plus reagent (Beyotime, Shanghai, China).

### Statistical Analysis

All data are presented as the mean ± standard deviation (SD). Between-group comparisons of the results were made by *t*-test (a *p*-value < 0.05 was considered statistically significant). Statistical analysis was performed and graphs were plotted by using GraphPad Prism 7.0 software.

## Results

### Quantitative Proteomic Analysis of Brain Tissues in Rats Showed That 282 DEPs Were Associated With Acute IS

**Figure 2:**
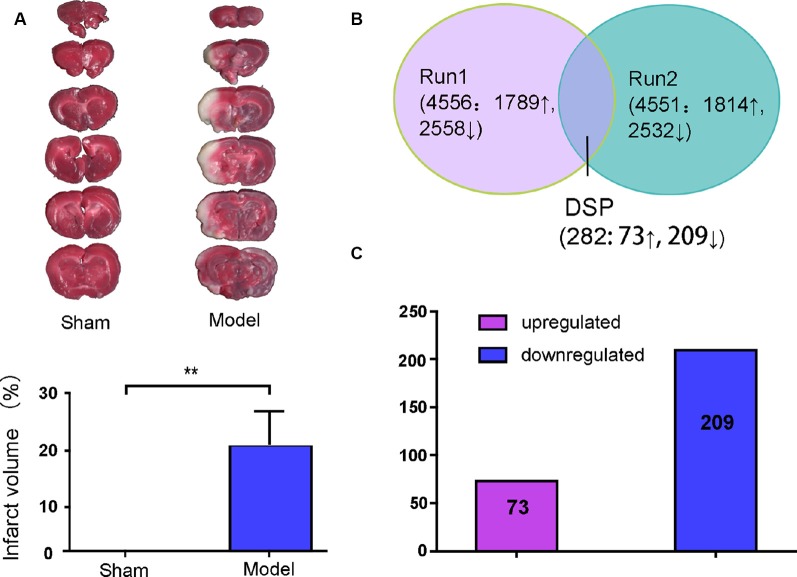
Brain infarction measurement and summary of the identified proteins displayed by Venn diagram and bar chart. **(A)** Middle cerebral artery occlusion (MCAO) induced a white area, which represents infarction, unlike the sham group. **(B)** Total proteins and overlap differentially expressed proteins (DEPs) presented in a Venn diagram. Significant changes in the protein ratio were set at 1.2-fold. **(C)** The average values of 282 DEPs with upregulated (purple) and downregulated (blue) expression between the model group and sham group. Numbers in rectangles respectively show the amounts of altered protein expression. **p < 0.01 compared to the sham group.

White infarction areas were observed in the model (MACO) group, while the sham group displayed uniform red staining. This means that the operation of the MACO model was successfully performed (Figure 2A). Protein extracts were exerted by the iTRAQ analysis from rat brains with or without acute IS. The 4578 proteins were identified based on FDR ≤1%. Only the regulated proteins with fold change ≥ 1.2 or with an average ratio change ≤0.83 as well as peptides (95%) ≥2 and unused value ≥2 in both replicates were considered as significantly expressed proteins. We found that there were 282 DEPs (73 upregulated and 209 downregulated; Figures 2B,C).

### Ontology and Functional Analysis of Acute IS-Associated DEPs

**Figure 3:**
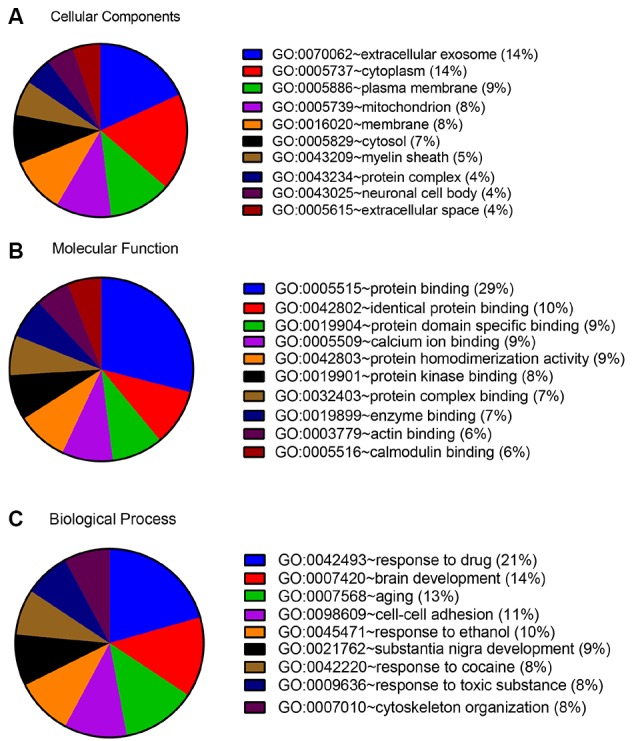
GO annotation analysis showed that acute ischemic stroke (IS) mainly involves exosome components and binding response to drug. **(A)** GO annotation by DAVID summarizing cellular components. **(B)** GO annotation summarizing molecular function. **(C)** GO annotation summary according to biological process.

**Figure 4:**
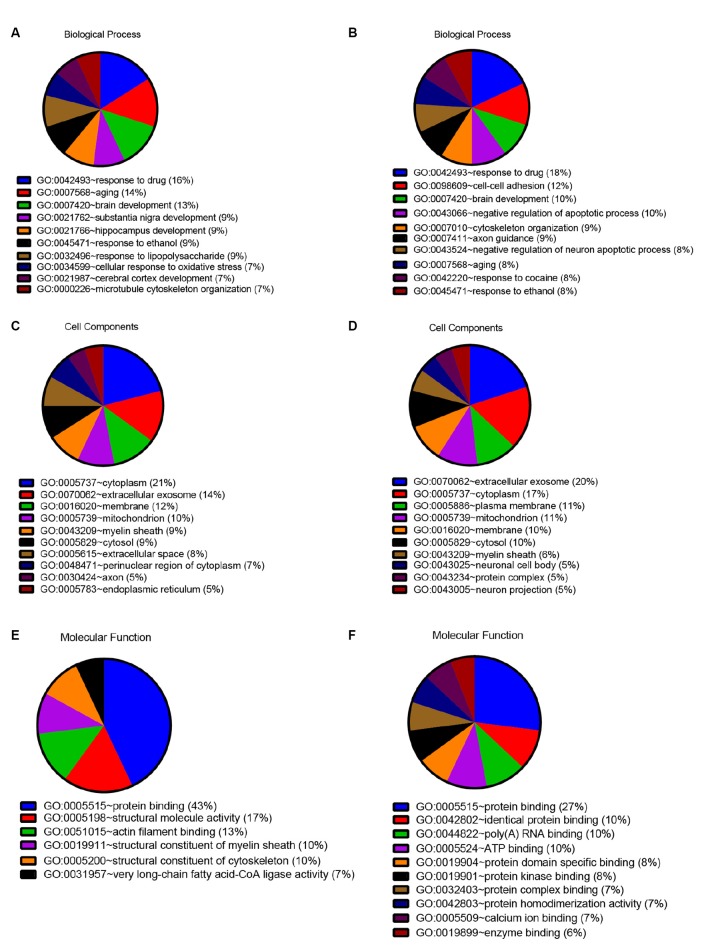
Quantitative GO annotation analysis in terms of upregulated and downregulated DEPs. GO analysis showed that the acute IS proteome mainly involves protein binding and response to drug for both upregulated and downregulated DEPs, while the cell component analysis showed the dominance of cytoplasm for upregulated DEPs and extracellular exosome for downregulated DEPs. **(A)** GO annotation by David summarizing biological process for upregulated DEPs. **(B)** GO annotation by David summarizing biological process for downregulated DEPs. **(C)** GO annotation by David summarizing cellular components for upregulated DEPs. **(D)** GO annotation by David summarizing cellular components for downregulated DEPs. **(E)** GO annotation summary according to molecular function for upregulated DEPs. **(F)** GO annotation summary according to molecular function for downregulated DEPs.

In order to detect the potential role of acute IS-related proteins, the 282 DEPs were examined through the DAVID platform with reference to the GO database. As shown in Figure 3A, the cellular components category of GO analysis for the proteins included extracellular exosome (14%), cytoplasm (14%), plasma membrane (9%), mitochondria (8%), membrane (8%), and cytosol (7%), which suggested the major types of cell localization. Protein binding was mainly significantly enriched in the molecular functional category, which is shown in Figure 3B. The top molecular functions include calcium ion protein binding (9%), protein homodimerization activity (9%), protein kinase binding (8%), and protein complex binding (7%). The biological process category of GO analysis shortlisted drug response (21%) and brain development (14%), followed by aging (13%), cell-cell adhesion (11%), response to ethanol (10%), and substantia nigra development (8%; Figure 3C). Notably, GO bioinformatics analysis suggested that the process of acute IS was mainly associated with potential pathophysiological biomarkers of extracellular secretion and cytoplasm, as well as of the pathological functions of the protein binding. In addition, Figure 4 shows upregulated and downregulated DEP separately according to GO bioinformatics analysis. The results mainly demonstrate that cytoplasm was implied in upregulated DEPs and that extracellular exosome was implied in downregulated DEPs.

### Pathway Database Analysis of Acute IS-Associated DEPs

**Figure 5:**
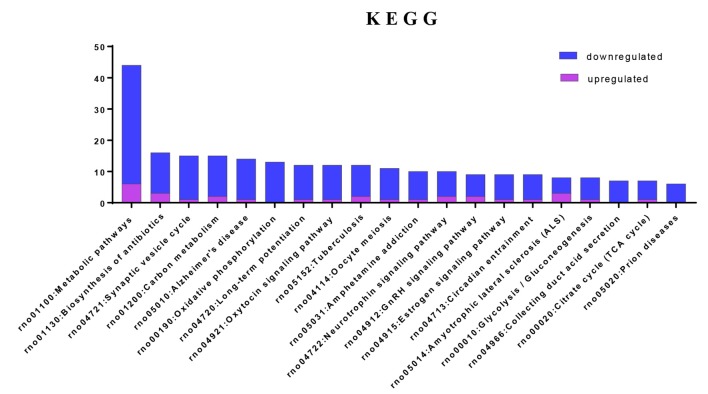
Enriched KEGG pathway analysis. Top 20 pathways were statistically siginificant with *p*-values < 0.05.

To investigate the important pathways by differential proteins in this study, 282 differential proteins were mapped to the KEGG database in DAVID. In particular, the background of the rat protein database was used for a DAVID functional annotation. As shown in Figure 5 and Table 1, the 20 identified pathways display high counts for protein abundance. The results illustrate that most of the differentiated proteins were significantly downregulated on the basis of enrichment pathway. Furthermore, the category “metabolic pathway” was the most markedly enriched in differentiated protein abundance. These metabolic pathways mainly contained carbon metabolism (15 related proteins), phosphorylation (13 proteins), and glycolysis (eight proteins). Interestingly, a number of DEPs that responded to acute IS were assessed to induce the coverage of the pathways in preconcentration of neurodegenerative diseases, including Alzheimer’s disease, Amyotrophic lateral sclerosis (ALS), and prion diseases. These degenerative diseases were associated with the pathways caused by neurological dysfunction and death. Collectively, the bioinformatics analysis may suggest that acute IS was mainly involved in essential pathophysiological pathways related to drug inflammation, neurological dysfunction, and metabolic processing.

**Table 1:** KEGG pathways associated by DAVID with acute IS.

Term	Count	*P*-value
rno01100:Metabolic pathways	44	0.000261
rno01130:Biosynthesis of antibiotics	16	0.0000263
rno04721:Synaptic vesicle cycle	15	0.00000000001
rno01200:Carbon metabolism	15	0.0000000958
rno05010:Alzheimer’s disease	14	0.0000602
rno00190:Oxidative phosphorylation	13	0.0000258
rno04720:Long-term potentiation	12	0.0000000456
rno04921:Oxytocin signaling pathway	12	0.000315
rno05152:Tuberculosis	12	0.000935
rno04114:Oocyte meiosis	11	0.0000548
rno05031:Amphetamine addiction	10	0.00000401
rno04722:Neurotrophin signaling pathway	10	0.000846
rno04912:GnRH signaling pathway	9	0.000448
rno04915:Estrogen signaling pathway	9	0.000597
rno04713:Circadian entrainment	9	0.00064
rno05014:Amyotrophic lateral sclerosis (ALS)	8	0.000095
rno00010:Glycolysis/Gluconeogenesis	8	0.000438
rno04966:Collecting duct acid secretion	7	0.0000115
rno00020:Citrate cycle (TCA cycle)	7	0.0000323
rno05020:Prion diseases	6	0.000372

### Network Analysis of Protein-Protein Interaction (PPI)

**Figure 6:**
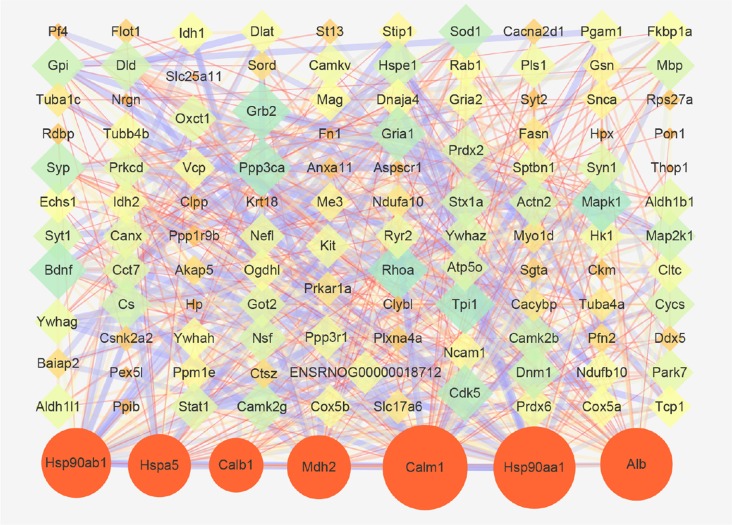
Protein-protein interaction (PPI) network of proteins that are differentially expressed in acute IS. The network was generated using the STRING database. There was an interaction network of DEP members. Six key proteins with a high degree are indicated by orange circles. Nodes represent the proteins, with the node size proportional to the interaction amount. Edges share PPIs, with the edge size proportional to the relationship.

**Figure 7:**
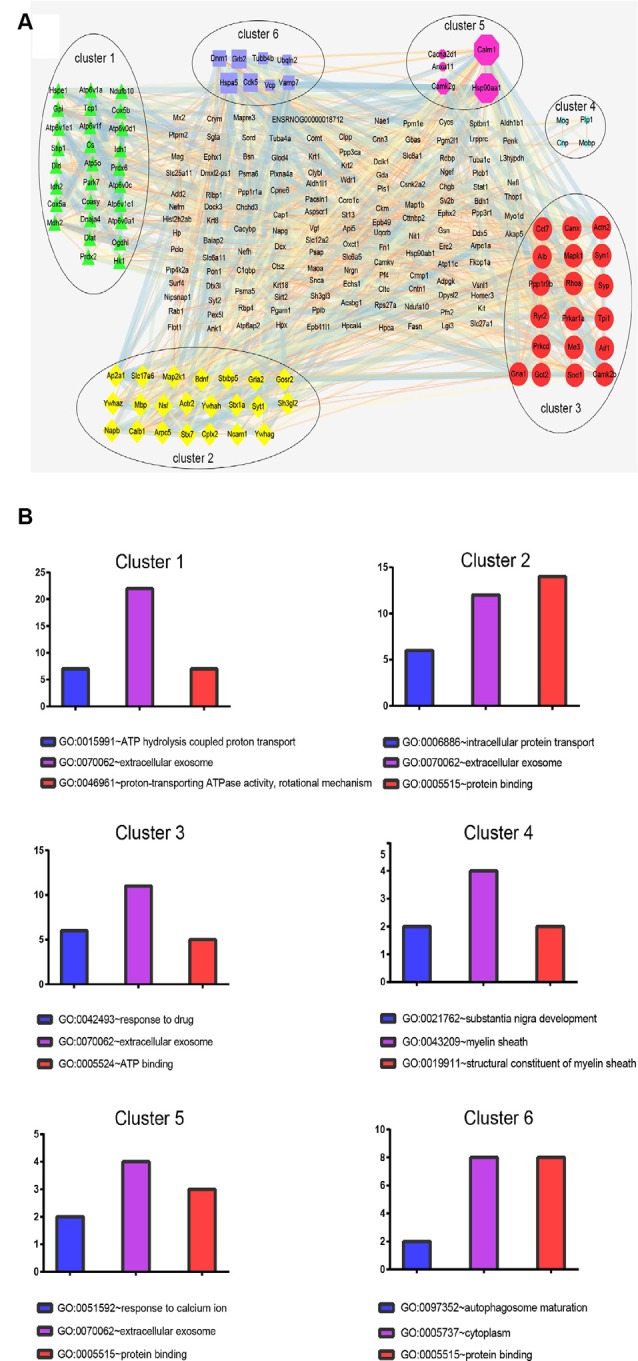
Protein cluster analysis with MCODE to find highly interconnected subnetworks involved in acute IS. **(A)** The six clusters in the PPI network are labeled with six oval shapes. **(B)** Statistical summary of the GO terms associated with the six clusters of DEPs, represented in bar charts. Data represent GO terms with the highest numbers of proteins. Bar color highlights the GO terms: blue, biological process; purple, cellular components; and red, molecular function. Y-axis represents the number of DEP candidates.

Next, a PPI network provided information on biological processes through the interactions between the identified proteins and their possible regulatory effects (Figure 6). Figure 6 showed that six of the proteins, namely CALM1, HSP90AA1, ALB, HSP90AB1, MDH2, and HSPA5, with a high degree in the PPI network, were the central proteins. Moreover, these six highly connected clusters were the major PPI subnetworks associated with acute IS (Figure 7A). By GO enrichment analysis, the potential relationships between these proteins and the predicted associations with biological processes in these six highly ranked subnetworks are expressed in Figure 7B. The results represent that the cellular component is mainly associated with exocrine and cell processes. These networks may provide us with overall protein interaction networks in an active process, which plays an important role in the regulatory mechanisms of acute IS.

### Western Blotting Validation

**Figure 8:**
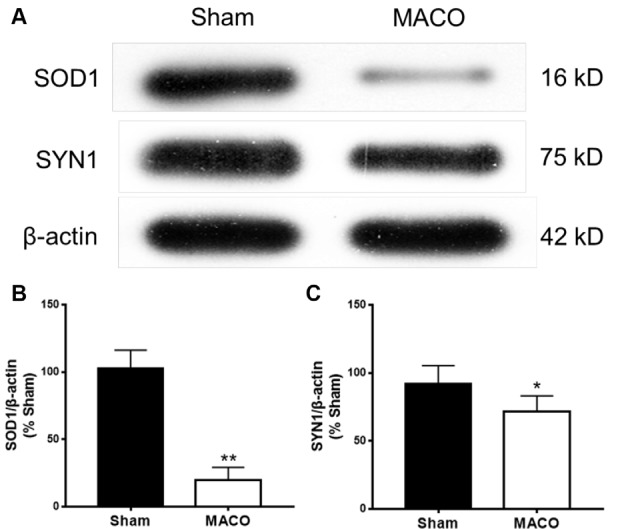
Expressions of SOD1 and SYN1 from brain tissues were examined by Western blotting. **(A)** Typical images of SOD1 and SYN1 proteins. **(B)** The statistical quantification showed that MACO significantly decreased the SOD1 level. **(C)** The statistical quantification showed that MACO significantly decreased the SYN1 level. *p < 0.05 and **p < 0.01 compared to the sham group.

To validate the results of the iTRAQ testing, we performed Western blotting to examine the relative contents of key functional proteins (SOD1 and SYN1) in the acute period of IS (Figure 8). SOD1 is related to neurodegeneration and SYN1 related to synaptic plasticity. Figure 8 suggested that, compared with the Sham group, the expressions of SOD1 and SYN1 were markedly downregulated in the MACO group, which was in accordance with the results of iTRAQ analysis.

## Discussion

The present study aims to investigate the proteomic profiles of brains from a rat model of acute IS. We identified 4,578 proteins through the iTRAQ-based proteomics method. Of these proteins, 282 differentiated proteins, comprising 73 upregulated and 209 downregulated proteins, were observed. Further bioinformatics analysis to reveal global protein profiles suggests that the 282 candidate proteins were mainly involved in energy liberation, intracellular protein transport, and synaptic plasticity regulation during the acute period in brains of IS rats. The KEGG pathway enrichment analysis indicates that a series of representative pathological pathways including energy metabolite, long-term potentiation (LTP), and neurodegenerative disease-related pathways are the top-ranked. Moreover, Western blotting verified the associated candidate proteins that refer to oxidative responses and synaptic plasticity. This study may offer a combination panel plus biological insights as an effective and valuable reference for the pathophysiology of acute IS.

Previous studies mainly focus on blood-based proteins to gain insights into the pathological mechanism of IS. However, due to the existence of the BBB, potential indexes from peripheral blood do not tend to truly respond to the pathophysiological process in the brain during IS. Thus, besides blood protein measurement, there is an increasing need to search for potential biomarkers from brain tissues (Ning et al., 2011; Chen et al., 2015; Lind et al., 2015; Smith and Gerszten, 2017; Muñoz et al., 2018; Simats et al., 2018). To address this concern, we used iTRAQ-labeled proteomics combined with LC-MS/MS and bioinformatics to screen multiple biomarkers and pathways in this study. These procedures unveil a global and systemic spectrum of proteins. This may help to describe the brain-based proteomics profiles that are responsible for the pathophysiology of acute IS.

According to GO terms analysis, we showed that the cellular component of extracellular secretions, the molecular function of protein binding, and the biological process of drug response gained the highest counts. Interestingly, extracellular exosome became a predominant category in brain according to our proteome cellular component analysis. This finding suggests that exosome-mediated regulation may perform a great role in the underlying pathogenetic mechanisms of acute IS. In fact, previous studies have documented that ischemic-simulated exosomes such as endogenous exosome secretion (e.g., HSP70) improve ischemic tolerance by preconditioning ischemic brain (Vega et al., 2008; Feng et al., 2014; Shevtsov et al., 2014; Kim et al., 2018). The extracellular membrane vesicles, including FN1, MAPK1, and PSDP, promote the therapeutic effects on brain repair as specialized cargoes of the stem cell secretome (Kim et al., 2012; Drago et al., 2013; Bruntz et al., 2014). Moreover, exsomes may induce different physiological and pathophysiological processes in a host through mediated communication (Boelens et al., 2014; Schorey et al., 2015; Zappulli et al., 2016; Raab-Traub and Dittmer, 2017; Huang and Deng, 2019). For example, damaged nerve tissues including microglia and astrocytes have been shown to release exosome, which carried inflammatory factors such as interleukin 1 to increase extracellular ATP (Fauré et al., 2006). Furthermore, the glycosylphosphatidyl-inositol-anchored (GPI-anchored) prion protein released by exosome potentiates a physiological response in neurons cultured from embryos by regulating its synaptic activity (Wei et al., 2019). Based on the above studies, exosomes may be candidates for indicating the pathophysiological process of acute IS, not only as potential biomarkers but also due to playing important roles in neural damage and microgila activation. These may indicate promising prognostic biomarkers and offer assistance in the therapeutic approach for IS.

Furthermore, KEGG analysis displays that the acute IS process significantly enriches pathways including metabolic pathways, biosynthesis of antibiotics, the synaptic vesicle cycle, and Alzheimer’s disease. We also find that carbon metabolism, with 15 enriched proteins, is the main metabolic pathway. It contributes to complex biological processes that suppress neuroinflammation, induction of hypoxia, and apoptosis, which is consistent with previous studies (Dong et al., 2017; Fedorovich et al., 2017; Correa-Costa et al., 2018). Our analysis reveals that most of the proteins were downregulated in these pathways, implying their important role in binding, metabolism and brain development, response to drug, and the immune system. These results may provide reference data for the pathogenesis, diagnosis, and drug therapy of acute IS.

In addition, significantly decreased levels were found for six key proteins, namely CALM1, HSP90AA1, ALB, HSP90AB1, MDH2, and HSPA5, which were described as central nodes in the disorder manifestation of acute IS. CALM1 may be important for its central roles in activating immune responses and responses to acute inflammation through harmful calcium overload in the damage progression of the ischemic brain (Li et al., 2016; Tsigelny et al., 2016; Yuan et al., 2016). Hsp90s (e.g., HSP90ab1 and HSP90aa1) are functionally identified in cellular stabilization, regulation, and protein activation (Richter et al., 2008; Huang et al., 2014). HSPA5 is involved in endoplasmic reticulum stress-induced apoptosis, which is one of the underlying mechanisms of HSPA5-mediated neuroprotection (Wang et al., 2015). The increased level of HSPA5 is related to infraction attenuation in focal ischemia-stroked mice (Tranter et al., 2010). An increased Alb level plays a role in the breakage of the BBB in CIR-induced brain damage (Chen et al., 2015). The downregulated MDH2 may be responsible for the failure of energy metabolism in brain infarct regions through the metabolic in-coordination between cytosol and mitochondria (Datta et al., 2013). Collectively, the position of these six downregulated central hubs in our predicted PPI network suggest that the dysregulation of these proteins and enzymes is in response to a series of biological function modulations in the pathophysiological process of IS. Thus, we may gain further biological insights into the acute IS process. Meanwhile, these hub components could be used as novel biomarkers. Their overlapping pathways should be determined as the main biological processes. Further studies on the regulations of multi-target and multi-pathway mechanisms in IS brain disease should be carried out.

Interestingly, the results show that exosomes are the main component. This fact may also suggest the importance of exosome in the acute IS process, which is consistent with our GO analysis. Notably, the central protein MDH2 in the PPI network is shown in the top cluster. The significant process for cluster 1 is the regulation of ATP hydrolysis coupled proton transport. The dysregulated TCA cycle reveals a failure of the cellular energy metabolism in IS (Kim and Baik, 2019). Meantime, MDH2 may result in the failure of energy metabolism through metabolic non-coordination between cytosol and mitochondria (Datta et al., 2013). Thus, these results imply that energy metabolism failure by dysregulation of the TCA cycle may play a main role in acute IS-related mechanisms.

To validate the results of the iTRAQ test, the Western blotting method was used to examine the relative contents of two key function proteins (SOD1, SYN1) after acute IS. According to iTRAQ analysis and the Western blotting validation, SOD1 and SYN1 were downregulated after acute IS. The dysregulation of SOD1 affects intracellular reactive oxygen species and leads to cellular oxidization damage in IS brain (Davis and Pennypacker, 2017). Additionally, SOD1 is the most frequent disease factor in ALS, where it gives rise to mutations (Picher-Martel et al., 2016). Neuronal aggregates of misfolded SOD1 protein may have prion-like properties and cause a fulminant ALS-like phenotype (Bidhendi et al., 2016). According to cellular component analysis, the validation of SOD1 may facilitate the understanding of the relationships between common neurodegenerative diseases such as IS. These may highlight potential new avenues for treating brain diseases.

SYN1 plays an important role in the synaptic transmission and plasticity associated with LTP, learning, and presynaptic plasticity (Vara et al., 2009; Cesca et al., 2010; Farisello et al., 2013). In cerebral ischemic post-conditioning, the expression of SYN1 may take part in the synaptogenesis associated with the recovery of behavioral function, a finding similar to the results of our analysis in this study (Wang et al., 2018). Thus, our study implies the role of SYN1 in the synaptic process following IS. Targeting SYN 1 may be a potentially crucial method related to synaptic plasticity recovery for treating IS.

We also should consider the insufficiency of MCAO. The insufficiency of MCAO is that it is impossible to directly determine whether the blood flow is cut off during the operation. Furthermore, the skill of the operator determines the success rate. We hope to analyze the clinical proteomics in a further study. In addition, because proteins from ischemic penumbra were collected in this study, there was potential bias in the proteomics compared with data analysis for the infarct zone. Further proteomics investigation of the core ischemic region should be implemented.

## Conclusion

In conclusion, an iTRAQ-labeled proteomics approach coupled with bioinformatics analysis demonstrated that acute IS caused significant changes in cerebral proteins that refer to underlying potential pathophysiological pathways. Our study may facilitate the identification of candidate protein biomarkers and provide insight into the biological processes involved in IS.

## Data Availability Statement

The datasets generated for this study can be found in the PRIDE, PXD006437.

## Ethics Statement

The animal study was reviewed and approved by Xiangya Hospital of Central South University For Animal Research Committee. Written informed consent was obtained from the owners for the participation of their animals in this study.

## Author Contributions

YW, TT, and J-KL conceived and designed experiments. YW, TL, and Z-YY performed most experiments. FZ, Y-FZ, Y-TZ, and YW implemented methodology and software analysis. FZ wrote the final draft. FZ, Y-TZ, YW, TT, and J-KL contributed to editing and approving the final manuscript.

## Conflict of Interest

The authors declare that the research was conducted in the absence of any commercial or financial relationships that could be construed as a potential conflict of interest.

## Footnotes

^1^https://david.ncifcrf.gov/

## References

[B1] Adams H. P. Jr. Bendixen B. H. Kappelle L. J. Biller J. Love B. B. Gordon D. L.. (1993). Classification of subtype of acute ischemic Stroke. Definitions for use in a multicenter clinical trial. TOAST. Trial of Org 10172 in acute stroke treatment. Stroke 24, 35–41. 10.1161/01.str.24.1.35 7678184

[B2] Bidhendi E. E. Bergh J. Zetterström P. Andersen P. M. Marklund S. L. Brännström T. (2016). Two superoxide dismutase prion strains transmit amyotrophic lateral sclerosis-like disease. J. Clin. Invest. 126, 2249–2253. 10.1172/JCI84360 27140399 PMC4887173

[B3] Boelens M. C. Wu T. J. Nabet B. Y. Xu B. Qiu Y. Yoon T.. (2014). Exosome transfer from stromal to breast cancer cells regulates therapy resistance pathways. Cell 159, 499–513. 10.1016/j.cell.2014.09.051 25417103 PMC4283810

[B4] Bruntz R. C. Lindsley C. W. Brown H. A. (2014). Phospholipase D signaling pathways and phosphatidic acid as therapeutic targets in cancer. Pharmacol. Rev. 66, 1033–1079. 10.1124/pr.114.009217 25244928 PMC4180337

[B5] Catanese L. Tarsia J. Fisher M. (2017). Acute ischemic stroke therapy overview. Circ. Res. 120, 541–558. 10.1161/CIRCRESAHA.116.309278 28154103

[B6] Cesca F. Baldelli P. Valtorta F. Benfenati F. (2010). The synapsins: key actors of synapse function and plasticity. Prog. Neurobiol. 91, 313–348. 10.1016/j.pneurobio.2010.04.006 20438797

[B7] Cevik O. Baykal A. T. Sener A. (2016). Platelets proteomic profiles of acute ischemic stroke patients. PLoS One 11:e0158287. 10.1371/journal.pone.0158287 27336623 PMC4919045

[B8] Chen H. J. Shen Y. C. Shiao Y. J. Liou K. T. Hsu W. H. Hsieh P. H.. (2015). Multiplex brain proteomic analysis revealed the molecular therapeutic effects of buyang huanwu decoction on cerebral ischemic stroke mice. PLoS One 10:e0140823. 10.1371/journal.pone.0140823 26492191 PMC4619651

[B10] Chen W. H. Yeh H. L. Tsao C. W. Lien L. M. Chiwaya A. Alizargar J.. (2018). Plasma translocator protein levels and outcomes of acute ischemic stroke: a pilot study. Dis. Markers 2018:9831079. 10.1155/2018/9831079 30034558 PMC6033241

[B11] Correa-Costa M. Gallo D. Csizmadia E. Gomperts E. Lieberum J. L. Hauser C. J.. (2018). Carbon monoxide protects the kidney through the central circadian clock and CD39. Proc. Natl. Acad. Sci. U S A 115, E2302–E2310. 10.1073/pnas.1716747115 29463714 PMC5877926

[B12] Datta A. Akatsu H. Heese K. Sze S. K. (2013). Quantitative clinical proteomic study of autopsied human infarcted brain specimens to elucidate the deregulated pathways in ischemic stroke pathology. J. Proteomics 91, 556–568. 10.1016/j.jprot.2013.08.017 24007662

[B13] Davis S. M. Pennypacker K. R. (2017). Targeting antioxidant enzyme expression as a therapeutic strategy for ischemic stroke. Neurochem. Int. 107, 23–32. 10.1016/j.neuint.2016.12.007 28043837 PMC5461189

[B14] Devaux Y. (2017). Transcriptome of blood cells as a reservoir of cardiovascular biomarkers. Biochim. Biophys. Acta Mol. Cell Res. 1864, 209–216. 10.1016/j.bbamcr.2016.11.005 27836747

[B15] Dong X. H. Peng C. Zhang Y. Y. Tao Y. L. Tao X. Zhang C.. (2017). Chronic exposure to subtherapeutic antibiotics aggravates ischemic stroke outcome in mice. EBioMedicine 24, 116–126. 10.1016/j.ebiom.2017.09.002 28928014 PMC5652002

[B16] Drago D. Cossetti C. Iraci N. Gaude E. Musco G. Bachi A.. (2013). The stem cell secretome and its role in brain repair. Biochimie 95, 2271–2285. 10.1016/j.biochi.2013.06.020 23827856 PMC4061727

[B17] Farisello P. Boido D. Nieus T. Medrihan L. Cesca F. Valtorta F.. (2013). Synaptic and extrasynaptic origin of the excitation/inhibition imbalance in the hippocampus of synapsin I/II/III knockout mice. Cereb. Cortex 23, 581–593. 10.1093/cercor/bhs041 22368083

[B18] Fauré J. Lachenal G. Court M. Hirrlinger J. Chatellard-Causse C. Blot B.. (2006). Exosomes are released by cultured cortical neurones. Mol. Cell. Neurosci. 31, 642–648. 10.1016/j.mcn.2005.12.003 16446100

[B19] Fedorovich S. Hofmeijer J. van Putten M. J. le Feber J. (2017). Reduced synaptic vesicle recycling during hypoxia in cultured cortical neurons. Front. Cell. Neurosci. 11:32. 10.3389/fncel.2017.00032 28261063 PMC5311063

[B20] Feng Y. Huang W. Meng W. Jegga A. G. Wang Y. Cai W.. (2014). Heat shock improves Sca-1^+^ stem cell survival and directs ischemic cardiomyocytes toward a prosurvival phenotype *via* exosomal transfer: a critical role for HSF1/miR-34a/HSP70 pathway. Stem Cells 32, 462–472. 10.1002/stem.1571 24123326 PMC5517317

[B22] Huang T. Deng C. X. (2019). Current progresses of exosomes as cancer diagnostic and prognostic biomarkers. Int. J. Biol. Sci. 15, 1–11. 10.7150/ijbs.27796 30662342 PMC6329932

[B21] Huang S. Monaghan J. Zhong X. Lin L. Sun T. Dong O. X.. (2014). HSP90s are required for NLR immune receptor accumulation in Arabidopsis. Plant J. 79, 427–439. 10.1111/tpj.12573 24889324

[B23] Kim A. Y. Baik E. J. (2019). Glutamate dehydrogenase as a neuroprotective target against neurodegeneration. Neurochem. Res. 44, 147–153. 10.1007/s11064-018-2467-1 29357018

[B24] Kim H. S. Choi D. Y. Yun S. J. Choi S. M. Kang J. W. Jung J. W.. (2012). Proteomic analysis of microvesicles derived from human mesenchymal stem cells. J. Proteome Res. 11, 839–849. 10.1021/pr200682z 22148876

[B25] Kim J. Y. Han Y. Lee J. E. Yenari M. A. (2018). The 70-kDa heat shock protein (Hsp70) as a therapeutic target for stroke. Expert Opin. Ther. Targets 22, 191–199. 10.1080/14728222.2018.1439477 29421932 PMC6059371

[B26] Laskowitz D. T. Kasner S. E. Saver J. Remmel K. S. Jauch E. C. Nowak R.. (2009). Clinical usefulness of a biomarker-based diagnostic test for acute stroke. The biomarker rapid assessment in ischemic injury (BRAIN) study. Stroke 40, 77–85. 10.1161/STROKEAHA.108.516377 18948614

[B27] Li L. Mo H. Zhang J. Zhou Y. Peng X. Luo X. (2016). The role of heat shock protein 90b1 in patients with polycystic ovary syndrome. PLoS One 11:e0152837. 10.1371/journal.pone.0152837 27046189 PMC4821534

[B28] Lind L. Siegbahn A. Lindahl B. Stenemo M. Sundström J. Ärnlöv J. (2015). Discovery of new risk markers for ischemic stroke using a novel targeted proteomics chip. Stroke 46, 3340–3347. 10.1161/strokeaha.115.010829 26542692

[B29] Lindsey M. L. Mayr M. Gomes A. V. Delles C. Arrell D. K. Murphy A. M.. (2015). Transformative impact of proteomics on cardiovascular health and disease: a scientific statement from the American heart association. Circulation 132, 852–872. 10.1161/CIR.0000000000000226 26195497

[B30] Liu S. Levine S. R. Winn H. R. (2010). Targeting ischemic penumbra Part I: from pathophysiology to therapeutic strategy. J. Exp. Stroke Transl. Med. 3, 47–55. 10.6030/1939-067x-3.1.47 20607107 PMC2896002

[B31] Makris K. Haliassos A. Chondrogianni M. Tsivgoulis G. (2018). Blood biomarkers in ischemic stroke: potential role and challenges in clinical practice and research. Crit. Rev. Clin. Lab. Sci. 55, 294–328. 10.1080/10408363.2018.1461190 29668333

[B390] Misra S. Kumar A. Kumar P. Yadav A. K. Mohania D. Pandit A. K.. (2017). Blood-based protein biomarkers for stroke differentiation: A systematic review. Proteom. Clin. Appl. 11, 1–11. 10.1002/prca.201700007 28452132

[B33] Muñoz R. Santamaría E. Rubio I. Ausín K. Ostolaza A. Labarga A.. (2018). Mass spectrometry-based proteomic profiling of thrombotic material obtained by endovascular thrombectomy in patients with ischemic stroke. Int. J. Mol. Sci. 19:E498. 10.3390/ijms19020498 29414888 PMC5855720

[B34] Ning M. Sarracino D. A. Kho A. T. Guo S. Lee S. R. Krastins B.. (2011). Proteomic temporal profile of human brain endothelium after oxidative stress. Stroke 42, 37–43. 10.1161/strokeaha.110.585703 21164131 PMC3696517

[B36] Picher-Martel V. Valdmanis P. N. Gould P. V. Julien J. P. Dupré N. (2016). From animal models to human disease: a genetic approach for personalized medicine in ALS. Acta Neuropathol. Commun. 4:70. 10.1186/s40478-016-0340-5 27400686 PMC4940869

[B37] Raab-Traub N. Dittmer D. P. (2017). Viral effects on the content and function of extracellular vesicles. Nat. Rev. Microbiol. 15, 559–572. 10.1038/nrmicro.2017.60 28649136 PMC5555775

[B38] Raffeld M. R. Debette S. Woo D. (2016). International stroke genetics consortium update. Stroke 47, 1144–1145. 10.1161/strokeaha.116.012682 26906919 PMC4919662

[B39] Rai A. J. Zhang Z. Rosenzweig J. Shih I. E. M. Pham T. Fung E. T.. (2002). Proteomic approaches to tumor marker discovery. Arch. Pathol. Lab. Med. 126, 1518–1526. 10.1043/0003-9985(2002)126<1518:PATTMD>2.0.CO;2 12456215

[B40] Richter K. Soroka J. Skalniak L. Leskovar A. Hessling M. Reinstein J.. (2008). Conserved conformational changes in the ATPase cycle of human Hsp90. J. Biol. Chem. 283, 17757–17765. 10.1074/jbc.M800540200 18400751

[B41] Schorey J. S. Cheng Y. Singh P. P. Smith V. L. (2015). Exosomes and other extracellular vesicles in host-pathogen interactions. EMBO Rep. 16, 24–43. 10.15252/embr.201439363 25488940 PMC4304727

[B42] Sharma R. Gowda H. Chavan S. Advani J. Kelkar D. Kumar G. S.. (2015). Proteomic signature of endothelial dysfunction identified in the serum of acute ischemic stroke patients by the iTRAQ-based LC-MS approach. J. Proteome Res. 14, 2466–2479. 10.1021/pr501324n 25807139

[B43] Shevtsov M. A. Nikolaev B. P. Yakovleva L. Y. Dobrodumov A. V. Dayneko A. S. Shmonin A. A.. (2014). Neurotherapeutic activity of the recombinant heat shock protein Hsp70 in a model of focal cerebral ischemia in rats. Drug Des. Devel. Ther. 8, 639–650. 10.2147/dddt.s62024 24920887 PMC4044995

[B44] Simats A. García-Berrocoso T. Ramiro L. Giralt D. Gill N. Penalba A.. (2018). Characterization of the rat cerebrospinal fluid proteome following acute cerebral ischemia using an aptamer-based proteomic technology. Sci. Rep. 8:7899. 10.1038/s41598-018-26237-3 29784938 PMC5962600

[B45] Smith J. G. Gerszten R. E. (2017). Emerging affinity-based proteomic technologies for large scale plasma profiling in cardiovascular disease. Circulation 135, 1651–1664. 10.1161/circulationaha.116.025446 28438806 PMC5555416

[B46] Sun D. Tiedt S. Yu B. Jian X. Gottesman R. F. Mosley T. H.. (2019). A prospective study of serum metabolites and risk of ischemic stroke. Neurology 92, e1890–e1898. 10.1212/WNL.0000000000007279 30867269 PMC6550501

[B47] Tranter M. Ren X. Forde T. Wilhide M. E. Chen J. Sartor M. A.. (2010). NF-κB driven cardioprotective gene programs; Hsp70.3 and cardioprotection after late ischemic preconditioning. J. Mol. Cell. Cardiol. 49, 664–672. 10.1016/j.yjmcc.2010.07.001 20643136 PMC3570030

[B48] Tsigelny I. F. Kouznetsova V. L. Lian N. Kesari S. (2016). Molecular mechanisms of OLIG2 transcription factor in brain cancer. Oncotarget 7, 53074–53101. 10.18632/oncotarget.10628 27447975 PMC5288170

[B49] Vara H. Onofri F. Benfenati F. Sassoè-Pognetto M. Giustetto M. (2009). ERK activation in axonal varicosities modulates presynaptic plasticity in the CA3 region of the hippocampus through synapsin I. Proc. Natl. Acad. Sci. U S A 106, 9872–9877. 10.1073/pnas.0900077106 19487674 PMC2701005

[B50] Vega V. L. Rodríguez-Silva M. Frey T. Gehrmann M. Diaz J. C. Steinem C.. (2008). Hsp70 translocates into the plasma membrane after stress and is released into the extracellular environment in a membrane-associated form that activates macrophages. J. Immunol. 180, 4299–4307. 10.4049/jimmunol.180.6.4299 18322243

[B51] Wang P. R. Wang J. S. Zhang C. Song X. F. Tian N. Kong L. Y. (2013). Huang-Lian-Jie-Du-Decotion induced protective autophagy against the injury of cerebral ischemia/reperfusion *via* MAPK-mTOR signaling pathway. J. Ethnopharmacol. 149, 270–280. 10.1016/j.jep.2013.06.035 23811213

[B52] Wang P. Zhang N. Liang J. Li J. Han S. Li J. (2015). Micro-RNA-30a regulates ischemia-induced cell death by targeting heat shock protein HSPA5 in primary cultured cortical neurons and mouse brain after stroke. J. Neurosci. Res. 93, 1756–1768. 10.1002/jnr.23637 26301516

[B53] Wang Y. Zhang Z. Zhang L. Yang H. (2018). ZRLIPostC protects against cerebral ischemia through improved synaptogenesis in rats. Brain Inj. 32, 1429–1436. 10.1080/02699052.2018.1483029 30036110

[B54] Wei C. J. Cui P. Li H. Lang W. J. Liu G. Y. Ma X. F. (2019). Shared genes between Alzheimer’s disease and ischemic stroke. CNS Neurosci. Ther. 25, 855–864. 10.1111/cns.13117 30859738 PMC6630005

[B55] Wesley U. V. Bhute V. J. Hatcher J. F. Palecek S. P. Dempsey R. J. (2019). Local and systemic metabolic alterations in brain, plasma and liver of rats in response to aging and ischemic stroke, as detected by nuclear magnetic resonance (NMR) spectroscopy. Neurochem. Int. 127, 113–124. 10.1016/j.neuint.2019.01.025 30707914

[B56] Whiteley W. Wardlaw J. Dennis M. Lowe G. Rumley A. Sattar N.. (2011). Blood biomarkers for the diagnosis of acute cerebrovascular diseases: a prospective cohort study. Cerebrovasc. Dis. 32, 141–147. 10.1159/000328517 21778711

[B57] Yang W. Sheng H. Thompson J. W. Zhao S. Wang L. Miao P.. (2014). Small ubiquitin-like modifier 3-modified proteome regulated by brain ischemia in novel small ubiquitin-like modifier transgenic mice: putative protective proteins/pathways. Stroke 45, 1115–1122. 10.1161/strokeaha.113.004315 24569813 PMC3966925

[B58] Yuan M. Tang Y. Zhou C. Liu F. Chen L. Yuan H. (2016). Elevated plasma CaM expression in patients with acute cerebral infarction predicts poor outcomes and is inversely associated with miR-26b expression. Int. J. Neurosci. 126, 408–414. 10.3109/00207454.2015.1020537 26001204

[B59] Zappulli V. Friis K. P. Fitzpatrick Z. Maguire C. A. Breakefield X. O. (2016). Extracellular vesicles and intercellular communication within the nervous system. J. Clin. Invest. 126, 1198–1207. 10.1172/jci81134 27035811 PMC4811121

[B60] Zhou F. Zhou L. Guo T. Wang N. Hao H. Zhou Y.. (2017). Plasma proteomics reveals coagulation, inflammation and metabolic shifts in H-type hypertension patients with and without acute ischemic stroke. Oncotarget 8, 100384–100395. 10.18632/oncotarget.22233 29245986 PMC5725028

[B61] Zhu W. Tian L. Yue X. Liu J. Fu Y. Yan Y. (2019). LncRNA expression profiling of ischemic stroke during the transition from the acute to subacute stage. Front. Neurol. 10:36. 10.3389/fneur.2019.00036 30774621 PMC6367239

